# Stream instabilities in optical-field ionization of a monatomic dilute neutral gas in fully relativistic regime

**DOI:** 10.1038/s41598-022-26815-6

**Published:** 2023-01-24

**Authors:** M. Ghorbanalilu

**Affiliations:** Departmetn of physics, Shahid Beheshti University, Tehran, 1983969411 Iran

**Keywords:** Fluid dynamics, Plasma physics, Physics

## Abstract

Stream instabilities arising from anisotropic electron velocity distribution function (EVDF) are discussed in the optical-field ionization mechanism of a monatomic dilute gas by a circularly polarized laser beam in a fully relativistic regime. It is shown that a relativistically rotating electron beam is derived by a circularly polarized laser field with ($p_z>p_\perp$). We show that the following ionization and before collisions thermalize the electrons, the plasma undergoes Buneman and Weibel instabilities. The Weibel and Buneman modes are co-propagating with *k* normal to the streaming direction. The theoretical results reveal that for the threshold of the relativistic regime ($a_0\approx 1$), instabilities are aperiodic and grow independently. However, by increasing the laser intensity for $a_0>1$, two instabilities are coupled. The coupling process increased the growth rate of Weibel instability, while the Buneman instability experienced a decrement in its growth rate. For more intense laser radiation, both instabilities are broken into different oscillatory and aperiodic modes.

## Introduction

The Weibel instability is a purely growing transverse electromagnetic wave in plasma, which is generated from the anisotropy of the electron velocity distribution function^1^. A very closely related instability was found by Fried^2^ that was driven by the momentum anisotropy and is usually referred as the current filamentation (CF). Although the CF instability is usually known with the Weibel instability, the filamentation mode is transverse provided both beams are strictly identical. The Weibel instability is thought to occur in fundamental phenomena such as gamma-ray burst^3^, collisionless shocks^4^, solar corona and interplanetary medium^5^, trapped solar wind in the ionosphere by Earth’s magnetic field^6,7^, neutrino-plasma interaction^8^ , quark-gluon plasmas^9,10^, microwave gas discharge^11,12^, optical breakdown^14,17,18,20^ , fast ignition scenario of initial confinement fusion^21^ and megagauss magnetic field generation in laser–plasma interaction^22,23^. In a sufficiently strong electric field, electrons move relative to motionless ions with a velocity much greater than their thermal velocity. Under this condition, a quickly growing aperiodic purely longitudinal instability occurs which is known as the Buneman instability. It can be thought of as a modified two-stream instability arising from the difference in drifts of electrons and ions^24^.

By progressing in intense ultrashort-pulse, ionization of atoms and/or molecules of gas has become possible in a few laser cycles. This process is known as optical field induce (OFI) or tunneling ionization. In the optical field induced OFI plasma initial electron velocity distribution function (EVDF) is highly anisotropic^14,17,18,20^, consequently, such kinds of plasmas can be the origin of the Weibel and Buneman instabilities^25^. The Buneman instability is electrostatic ($k\times E=0$), and generates longitudinal waves via inverse Landau damping. Also for symmetrical streams, Buneman instability is aperiodic. The both instabilities grow simultaneously in OFI plasmas. The existence of kinetic instabilities in plasma has already been discussed in theoretical work^26^ and relatively few laboratory verifications^17–19,27–31^. It is shown that Weibel instability in microwave-produced plasma is purely transverse ($k.E=0$) and aperiodic mode^11,12^, however, for the optical breakdown in weakly relativistic regime the Weibel instability is an oscillating mode (Weibel-like)^14^. In the recent investigation, the radial Weibel instability has been observed in relativistic intensity laser-plasma interactions inside a sub-micron thick liquid target^15^. Both the experimental and simulation results show the presence of strong magnetic fields characteristic of Weibel instability that is accompanied by radial expansion of plasma. Furthermore, steam instabilities in the relativistic ion beam interaction with hot plasma have already been discussed where revealing the coupling between Weibel and Buneman instabilities^16^.

Since the laser was invented, the peak power and focus ability continually increased. By advanced, laser system and using the focus technique, the intensities above $I\simeq 10^{22} \, \textrm{W} \, \textrm{cm}^{-2}$ are available, nowadays. In such intensities, an ionized state is abruptly created in the interaction between laser and matter. The electrons oscillate at relativistic velocities in the laser field and a giant electric current is produced. The produced electron beam during the optical-field ionization propagates through motionless ions in both perpendicular and parallel directions. Consequently, for a dilute gas for pressure $P\simeq 10$–100 torr, a weakly ionized non-equilibrium plasma is generated. However, in non-relativistic and weakly relativistic regimes the stability of such kind of plasmas have already been studied^11–14^ but we believe in fully relativistic regime the scenario is changed because of the rotation of plasma hot axis from normal to the parallel with the laser propagation direction compared to the weakly relativistic regime. This rotation changes the Weibel instability propagation direction and caused coupling of the Buneman and Weibel modes. In this paper, we offer a simple model to explain the optical-field ionization of a dilute gas by an intense circularly polarized laser beam. We derived the electron momentum distribution function due to tunneling ionization which shows a rotating relativistic electron beam moving through heavy immobile ions. We present the dispersion equation for perturbations normal to the streaming direction. The results show the excitation and coupling of the Weibel and Buneman instabilities for the relativistic regime ($a_0>1$).

## Simple model for optical field induced plasma

### Electron momentum distribution function and dielectric tensor elements in OFI plasmas

We assume a monochromatic circularly polarized laser field with a vector potential $A(\xi ) =\hat{e}_x A_0 \cos \xi +\hat{e}_yA_0\sin \xi$ in which $\xi =\omega _0(t-z/c)$ is the laser field phase where $A_0$ and $\omega _0$ are the maximum amplitude of the vector potential and laser field frequency, respectively. However, there is no a plane wave in reality and the temporal ponderomotive force can not be neglected, if we assume a slowly varying amplitude approximation this force is ignorable for the circularly polarized laser field. Since the gas is very dilute, collision is infrequent. On the other hand, the plasma density is very low and the polarization field is ignorable. Furthermore, the recombination process is not important. Under this condition, the kinetic equation for the electron originating by strong laser field in OFI plasmas is given by:

1 $$\begin{aligned}{} & {} \frac{\partial f_0}{\partial t}+\mathbf{v(r,t)}\frac{\partial f_0}{\partial r}+e\left[ \mathbf{E(r,t)}+\frac{1}{c}[\mathbf{v(r,t)}\times \mathbf{B(r,t)}]\right] \frac{\partial f_0}{{\partial p}}=S_{ioniz}\delta (p), \end{aligned}$$

where $f_0$ conveys to the electron momentum distribution function (EMDF) and $\mathbf{E(r,t)}=-(\partial \mathbf {A(r,t)}/c\partial t)$ and $\mathbf{B(r,t)}=\nabla \times \mathbf {A(r,t)}$ indicate the electric and magnetic fields of the radiation laser field. Furthermore, $S_{ioniz}$ defines the electron production rate per cubic centimeter with zero momentum. The solution of Eq. (1) by the method of characteristics leads us to the following result:

2 $$\begin{aligned} f_0(\mathbf{p'},\xi )=\frac{1}{\omega _0}\int d\xi _0(1+\frac{p_z}{m_0c})S_{ioniz}\delta (\textbf{p}'-\textbf{p}), \end{aligned}$$

where $\xi _0$ is the field phase at which the test electron originated and $\textbf{p}$ refers to the momentum of the electron, respectively, deduced by the solution of the Lorentz equation for electron motion in the laser field. By making use of the relativistic equation of motion for electron irradiated by a circularly polarized laser field and energy conservation equation $m_0c^2(d\gamma _{rel}/dt)=-e\textbf{E}.\textbf{v}$, we can show that $\tilde{p_z}=(\tilde{p_x}^2+\tilde{p_y}^2)/2$ and $\gamma _{rel}=1+\tilde{p_z}$ where $\gamma _{rel}$ is the relativistic Lorentz factor and $\tilde{p}$ is the momentum normalized to $m_0c$. By following the relativistic electron equation of motion, components of the electron’s momentum are given by^32^:

3 $$\begin{aligned}{} & {} p_x=-a_0m_0c(\cos \xi -\cos \xi _0),\nonumber \\{} & {} p_y=a_0m_0c(\sin \xi -\sin \xi _0) ,\nonumber \\{} & {} p_z=2a_0^2m_0c\sin ^2\left( \frac{\xi -\xi _0}{2}\right) , \end{aligned}$$

where $a_0=eA_0/m_0c^2$ is the normalized vector potential. We assume the electron originated with zero momentum at $\xi =\xi _0$ in deriving Eq. (3). The ionization of atoms by a strong laser field is usually modeled as a process in which an electron tunnels through the Coulomb barrier suppressed by the electric field. The model is valid when Keldesh parameter $\gamma '=(E_{ioniz}/2U_P)\ll 1$ is held, where $E_{ioniz}$ is the field-free ionization energy of a bound electron, and $U_p$ is the ponderomotive energy of interaction of a free electron with the strong laser field. If we neglect the processes such as recombination and or electron-impact ionization during the gas breakdown, we write the electron production rate as $S_{ioniz}=\displaystyle \sum _{k=0}^{z-1}w_kn_k,$ where $n_k$ and $w_k$ indicate the density of ion and probability of ionization in the *k*th ionization state, respectively, and *z* refers to the maximum ion charge number. When the laser field amplitude is increased smoothly, all ionization states of the gas atoms are ionized instantaneously. Consequentially, we can estimate the ionization probability in the form $w_k=\delta (t_0-t_{th}^k)=\omega _0\delta (\xi _0-\xi _{th}^k)$, where $t_{th}^k$ and $\xi _{th}^k$ are the threshold time for ionization of the atom in the *k*th state and the phase of laser field at this time. Using the Eqs. (2) and (3), we can obtain the EMDF in optical-field ionization of a monatomic gas as follow^11,12^

4 $$\begin{aligned} f_0(p_\bot ,p_z)=\frac{z\eta n_a\left( 1+\frac{p_z}{m_0c}\right) \delta \left( p_z-\beta \right) }{2\pi ^2p_\bot \sqrt{4a_0^2m_0^2c^2-p_\bot ^2}}, \end{aligned}$$

where $\beta =2a_0^2\sin ^2\varphi$ in which $\varphi =(\xi -\xi _{th}^k)/2$ and $p_\bot =\sqrt{p_x^2+p_y^2}$, $n_a$ is equilibrium plasma density and $\eta$ is the ratio of ionized atom to the neutral one. The Eq. (4) shows a highly anisotropic EMDF (i. e., $p_z>p_\perp$), with rotating electron beam propagating relativistically through immobile heavy ions. Such structure is susceptible to stream instabilities and for the time scale smaller than e-e collisions, we can turn to the adiabatic approximation and assume the instabilities grow faster than the plasma density. Therefore, we can use the dispersion relation $\Lambda _{ij}=0$ (where, $\Lambda _{ij}=|k^2\delta _{ij}-k_ik_j-\frac{\omega ^2}{c^2}\varepsilon _{ij}(\omega ,k)|$) for electron perturbations, where $\varepsilon _{ij}(\omega ,k)$ refers to the dielectric permittivity tensor corresponding to OFI plasma. The dielectric tensor is derived by linearizing the Vlasov equation and taking into account the self-consistency effect of Maxwell equations and cold ions approximation as^33^:

5 $$\begin{aligned}{} & {} \varepsilon _{ij}(\omega ,k)=\delta _{ij}-\frac{\omega _{pi}^2}{\omega ^2}\delta _{ij}+\frac{4\pi e^2}{\omega }\int dp\frac{v_i}{\omega -k.v}\frac{\partial f_0}{\partial p_j}, \end{aligned}$$

where $\omega _{pi}=(4\pi \eta z n_a e^2/M)^{1/2}$ and $\omega _{pe}=(4\pi \eta z n_a e^2/m_0)^{1/2}$ are ion and electron plasma frequencies in which *M* and $m_0$ refer to ion and electron masses, respectively. In plasma electrodynamic permittivity is the measure of electric polarizability and magnetization of a plasma. In laser-produced plasma, plasma permittivity is a tensor and a function of laser and plasma properties. The dielectric tensor elements are obtained by making use of EMDF and solving the residue integrals, carefully. By exploiting the cylindrical symmetry of the problem, we can assume with no loss of generality that $k_\perp =k_x$. The nonzero electron contribution of the dielectric permittivity can be obtained as follow

6 $$\begin{aligned}{} & {} \delta \varepsilon _{xx}^e =\frac{\omega _{pe}^2(1+\frac{\beta }{m_0c})}{\omega ^2\chi }\left( \frac{1}{\sqrt{1-\frac{4a_0^2c^2k_\bot ^2}{\omega ^2\chi ^2}}}+\frac{1}{\left( 1-\frac{4a_0^2c^2k_\bot ^2}{\omega ^2\chi ^2}\right) ^\frac{3}{2}}\right) ,\nonumber \\{} & {} \delta \varepsilon _{xz}^e=-\delta \varepsilon _{zx}^e =\frac{\omega _{pe}^2(1+\frac{\beta }{m_0c})\left( \frac{\omega }{k_\bot c}-\frac{k_z}{k_\bot }\right) }{\omega ^2\chi }\left( \frac{1}{\sqrt{1-\frac{4a_0^2c^2k_\bot ^2}{\omega ^2\chi ^2}}}-\frac{1}{\left( 1-\frac{4a_0^2c^2k_\bot ^2}{\omega ^2\chi ^2}\right) ^\frac{3}{2}}\right) ,\nonumber \\{} & {} \delta \varepsilon _{zz}^e=\frac{\omega _{pe}^2(1+\frac{\beta }{m_0c})}{\omega ^2\chi }\left( \frac{1}{\sqrt{1-\frac{4a_0^2c^2k_\bot ^2}{\omega ^2\chi ^2}}}-\frac{\left( \frac{k_\bot \beta }{m_0\omega }\right) \left( \frac{\omega }{k_\bot c}-\frac{k_z}{k_\bot }\right) }{\chi \left( 1-\frac{4a_0^2c^2k_\bot ^2}{\omega ^2\chi ^2}\right) ^\frac{3}{2}}\right) , \end{aligned}$$

where $\chi =1+(k_\bot \beta /m_0\omega )(\omega /ck_\bot -k_z/k_\bot )$. For fully relativistic regime (i. e., $\tilde{p_z}>\tilde{p_\perp }$), and for perturbations with *k* normal to the stream direction (i. e., $k_z=0$), we obtain

7 $$\begin{aligned}{} & {} <(1+\delta \varepsilon _{xx})>_\varphi \left<k^2-\frac{\omega ^2}{c^2}\left( 1+\delta \varepsilon _{zz}\right) \right>_\varphi +\frac{\omega ^2}{c^2}<\delta \varepsilon _{xz}\delta \varepsilon _{zx}>_\varphi =0, \end{aligned}$$

where the brackets denote averaging over $\varphi$.

**Figure 1:**
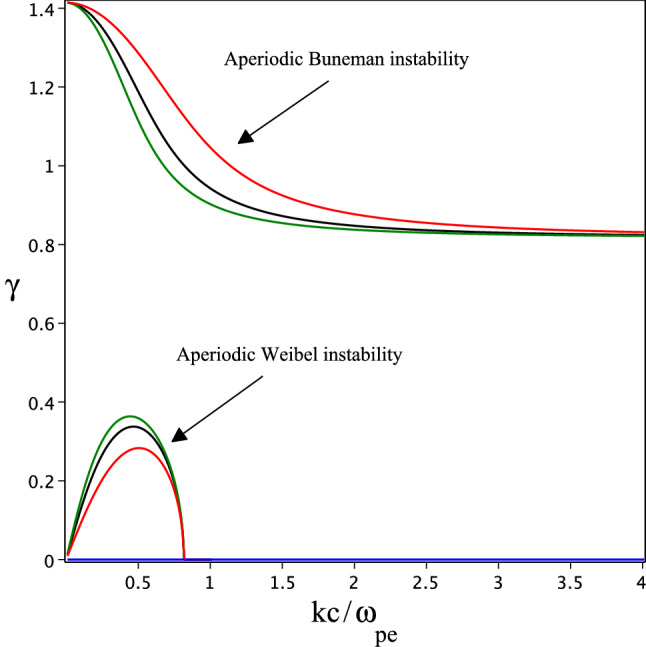
The growth rates of the Buneman and Weibel instabilities in the uncoupled limit, by the solution of the Eq. (8). Here $\gamma =Re\bar{\omega }$ for blue, and $\gamma =Im\bar{\omega }$ for red, black and green lines. The normalized amplitude of radiated laser field is $a_0=1,2,3$ for red, black, and green colors, respectively. We consider the ratio of the mass of an electron and ion as $m_0/M=1/1838$.

**Figure 2:**
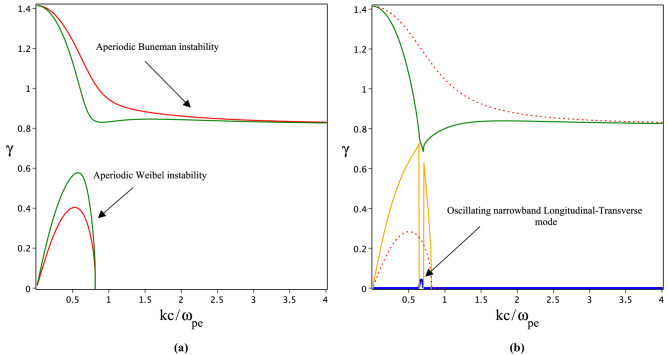
The growth rates of Longitudinal-Transverse instability in coupled limit. (**a**) Red and green lines indicate growth rates ($\gamma =Im\bar{\omega }$) of Weibel and Buneman instabilities in weakly coupled limit for $a_0=1,1.3$, respectively. (**b**) Yellow and green lines indicate growth rates ($\gamma =Im\bar{\omega }$) of Weibel and Buneman instabilities respectively for $a_0=1.39$, the blue line shows a small real part of modes frequencies at $kc/\omega _{pe}\approx 0.7$. The dot lines plotted instabilities growth rates in the uncoupled regime for $a_0=1$.

## Results

### Stability analyses of laser produced plasma in OFI mechanism

**Figure 3:**
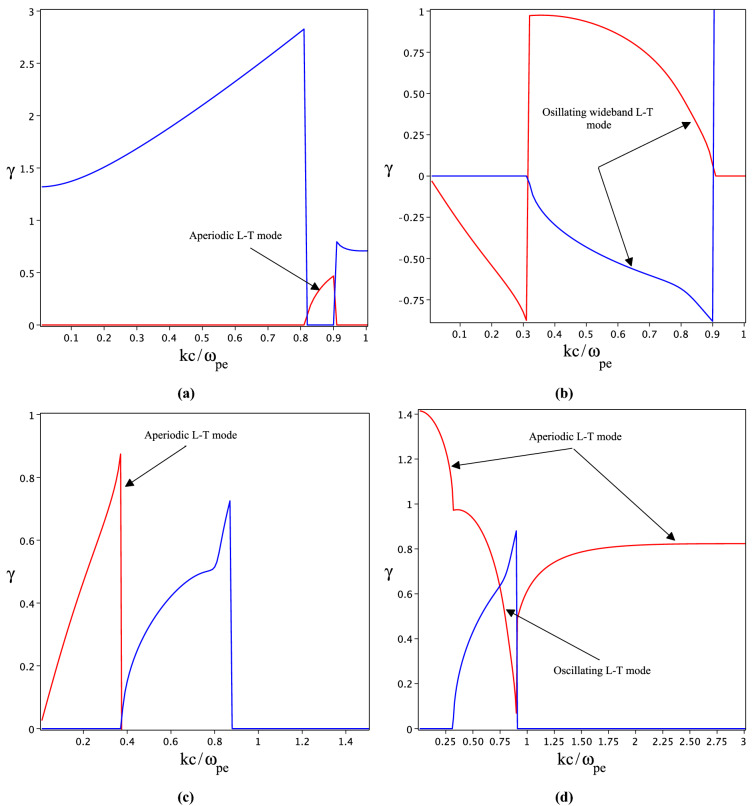
The spectrum of Longitudinal-Transverse instability in strongly coupled limit for $a_0=2$. The blue line indicates the real parts of mode frequency $\gamma =Re\bar{\omega }$ and the red line shows instability growth rate $\gamma =Im\bar{\omega }$. (**a**,**c**) The aperiodic and (**d**) shows oscillating modes. (**d**) Contains both oscillating and aperiodic modes at different frequencies ranges.

In the absence of the last term in Eq. (7), this equation shows dispersion relations for separated longitudinal and transverse waves. The last term cause to coupling between the longitudinal and transverse waves. So, we expect to generation of the new type of modes by solution of Eq. (7). Therefore, to better understanding how the modes coupling takes place, we solve this equation in the following limits.

#### Uncoupled limit

we assume the coupling term $<\delta \varepsilon _{xz}\delta \varepsilon _{zx}>_\varphi$ is ignorable. This condition is valid for the threshold of the relativistic regime ($a_0\thickapprox 1$). So, the dispersion equation is separated into two independent longitudinal and transverse dispersion equations. For longitudinal wave, we solve dispersion relation $<(1+\delta \varepsilon _{xx})>_\varphi =0$. By making use of the tensor elements Eq. (6), and for cold ion approximation, and after averaging over variable $\varphi$, we obtain

8 $$\begin{aligned} \bar{\omega }^4+\bar{\omega }^2\left( 2-\frac{m_0}{M}-\frac{6y^2a_0^2(a_0^2+1)}{(1+2a_0^2)^\frac{3}{2}}\right) -\frac{4y^2a_0^2(a_0^2+1)}{(1+2a_0^2)^\frac{3}{2}}=0, \end{aligned}$$

where $$\bar{\omega }=\omega /\omega _{pe}$$ and $$y=kc/\omega _{pe}$$.

Figure 1 shows the root of dispersion relation Eq. (8) with positive imaginary part, as a function of $kc/\omega _{pe}$. The aperiodic purely longitudinal instability arises by charge separation due to the motion of the relativistic electron through cold and immobile heavy ions in the transverse direction. We know this aperiodic instability as the Buneman instability. This mode is aperiodic because of the symmetric motion of electrons in the transverse direction. The figure shows that the instability growth rate decreases by increasing the laser intensity. Furthermore, instability can generate large-amplitude electrostatic wave at low frequency which is in good agreement with previous investigation on Buneman instability generation in laser-plasma interaction^34^. The dispersion relation for a purely transverse wave is obtained by dispersion equation $\left<k^2-\frac{\omega ^2}{c^2}\left( 1+\delta \varepsilon _{zz}\right) \right>_\varphi =0$, and using Eq. (6) as follow

9 $$\begin{aligned} \left( 1-\frac{m_0}{M}\right) \bar{\omega }^4-\bar{\omega }^2\left( 2+\frac{6a_0^2y^2}{\sqrt{1+2a_0^2}}+\frac{1-2a_0^2}{(1+2a_0^2)^\frac{3}{2}} \right) -\frac{4y^2a_0^2(a_0^2+1)}{(1+2a_0^2)^\frac{3}{2}}\left( 1-\frac{3}{2}y^2\right) =0. \end{aligned}$$

The aperiodic purely growing transverse mode in Fig. 1 is obtained by solution of the Eq. (9). We known this mode as Weibel instability. The figure clearly shows that the Weibel instability growth rate is increased by laser field strength. The Weibel instability depicted in Fig. 1 confirms the previous knowledge for Weibel instability in the relativistic plasmas^35^.

#### Coupled limit

By substituting tensor element Eq. (6) in dispersion Eq. (7) and averaging over variable $\varphi$, we obtain the dispersion relation for longitudinal–transverse (L–T) waves excited in OFI plasmas as follow

10 $$\begin{aligned}{} & {} \left( 1-\frac{m_0}{M}\right) \bar{\omega }^8+\bar{\omega }^6\left( (2-\alpha _3)-(1-\alpha )\alpha _1\right) -\bar{\omega }^4\left( \frac{3}{2}\alpha _3+\alpha _1(2-\alpha _3)-\left( 1-\frac{m_0}{M}\right) \alpha _2\right) \nonumber \\{} & {} +\bar{\omega }^2\left( (2-\alpha _3)\alpha _2-8\alpha _4+\frac{2}{3}\alpha _3\alpha _2\right) -\frac{2}{3}\alpha _3\alpha _2=0, \end{aligned}$$

where the coefficients $\alpha _1$, $\alpha _2$, $\alpha _3$ and $\alpha _4$ are given by

11 $$\begin{aligned}{} & {} \alpha _1=2+y^2-\frac{m_0}{M}+\frac{12a_0^4y^2+6a_0^2y^2-2a_0^2+1}{(2a_0^2+1)^\frac{3}{2}}, \quad \alpha _2=\frac{2a_0^2y^2(a_0^2+1)(3y^2-2)}{(2a_0^2+1)^\frac{3}{2}},\nonumber \\{} & {} \alpha _3=\frac{6a_0^2y^2(a_0^2+1)}{(2a_0^2+1)^\frac{3}{2}}, \quad \alpha _4=\frac{a_0^4y^2\left( 5a_0^6+9a_0^4+6a_0^2+2\right) }{(2a_0^2+1)^\frac{7}{2}}. \end{aligned}$$

The Eq. (10) shows the dispersion relation corresponding to the L-T waves that arise by the combined effects of electron momentum anisotropy and relativistic motion of electrons in the laser field. We solved this equation numerically and plotted the results in Figs. 2 and 3. Figure 2a is plotted for $a_0=1$ and $a_0=1.3$, respectively. The figure shows by increasing laser field amplitude, the Weibel and Buneman instabilities going to be effectively coupled. The growth rate of Weibel instability increased and the Buneman instability experienced a decrement in its growth rate while both instabilities remain aperiodic. Figure 2b indicates the coupled modes growth rate for $a_0=1.39$. The figure shows a new narrowband oscillating L–T mode which is generated in the region where Weibel and Buneman instability is strongly coupled. It is intuitively understood that, the modes strongly coupled when the growth rates are comparable. In contrast to the purely growing Weibel mode, the oscillatory Weibel mode propagates while growing. This means that the Weibel mode not only grows in place but can propagate in the plasma.

Figure 3 shows the growth rate of coupled modes for a more intense laser field with normalized amplitude $a_0=2$. It seems that the mode coupling takes place in a wide range of frequencies. Therefore, the wide band oscillating and narrow band aperiodic L–T modes are generated. For example in the coupling range of frequency $0<kc/\omega _{pe}<1$ Fig. 3a,c demonstrate aperiodic mode, while Fig. 3b indicates a wide band oscillating mode. In Fig. 3d, both oscillating and aperiodic modes are excited. Figure 3d clearly shows that the Buneman instability is suppressed by Weibel instability which is in agreement with the result reported for coupling of Weibel and Buneman instabilities in relativistic ion beam interaction with hot electrons background^16^.

## Discussion

In a non-relativistic regime $a_0\ll 1$, the originated electron picks up energy from the laser field along the polarization direction and the electron is hot in that direction ($p_\perp$). For weakly or fully relativistic regime ($a_0\le 1$ or $a_0>1$) electron is pushed in the forward and transverse directions and plasma usually has two momentum components ($p_\perp$ and $p_z$). Consequently, for a laser beam propagating in a parallel direction the conditions $p_\bot >p_z$ and $p_z>p_\perp$ are satisfied for weakly and fully relativistic regimes, respectively. In optical field ionization of a gas by a relativistic circularly polarized laser field the hot axis is along the laser field propagation direction ($p_z>p_\perp$). Such kind of anisotropic plasma is a source for excitation of stream instabilities. We shown that the Buneman and Weibel instabilities are excited and grow in OFI plasmas. In cylindrical coordinates, the electric and magnetic characteristics of Weibel and Buneman instabilities are given by ($k_r,B_\theta ,E_z$) and $(k_r,E_r)$, respectively. Streakily speaking, electrons experience two phenomena simultaneously, filamentation due to the Weibel instability and bunching because of Buneman instability. When the growth rates of instabilities get closer, coupling between modes takes place. By taking into account that the Weibel instability is increased by increasing anisotropy, for a high-relativistic regime we expect that filamentation overcomes the electron bunching. By comparison of Figs. 2a and 1 for $a_0=1$, it is revealed that the growth rate of Weibel/Buneman instabilities increases/decreases in the coupled limit. Although, as soon as the amplitude of the laser field increases to $a_0=1.3$ an effective coupling appears while both modes are still aperiodic. When the laser field amplitude increases to $a_0=1.39$ in Fig. 2b, the growth rates of both instabilities become comparable for $kc/\omega _{pe}\approx 0.7$ and two modes experienced a strong coupling. This coupling produces a new narrowband oscillating L–T mode. In Fig. 3, we plotted the spectrum of coupled modes for a more strong laser field $a_0=2$. As the figure shows, in the wide range of frequencies the growth rates of both instabilities are comparable, so the wideband oscillating L–T (Fig. 3b,d) and aperiodic L–T (Fig. 3a,c) modes are excited during OFI plasma. In summary, in the optical-field ionization of neutral gas in the fully relativistic regime, the Weibel instability produced by momentum anisotropy is not a purely transverse aperiodic mode. The Weibel mode is L–T and in the range of different frequencies is oscillating or aperiodic. This phenomenon arises by coupling purely transverse Weibel and purely longitudinal Buneman instabilities. Consequently, when the laser field intensity is in threshold value $a_0\approx 1$, the Buneman instability grows very fast and by increasing the laser field intensity by coupling modes the Buneman instability is suppressed by Weibel mode. The situation is very similar to the excited astrophysical instabilities in the beam-plasma interaction. When the beam density is low, the Buneman or two-stream instabilities are the governed modes, however, for beam density equal to plasma density the Weibel or filamentation modes overcome to the electrostatic modes^36^.

## Data Availability

The datasets generated during and/or analysed during the current study are available from the corresponding author on reasonable request.
